# Impact of the COVID-19 pandemic on the sexual behavior of the population. The vision of the east and the west

**DOI:** 10.1590/S1677-5538.IBJU.2020.S116

**Published:** 2020-07-27

**Authors:** François Peinado Ibarra, Mehri Mehrad, Marina Di Mauro, Maria Fernanda Peraza Godoy, Eduard García Cruz, Mohammad Ali Nilforoushzadeh, Giorgio Ivan Russo

**Affiliations:** 1 Hospital Quirón Salud Juan Bravo Department of Urology Madrid Spain Department of Urology, Hospital Quirón Salud Juan Bravo, Madrid, Spain; 2 Tehran University of Medical Sciences Skin and Stem Cell Research Center Tehran Iran Skin and Stem Cell Research Center, Tehran University of Medical Sciences, Tehran, Iran; 3 University of Catania Department of Surgery and Urology Section Catania Italy Department of Surgery and Urology Section, University of Catania, Catania, Italy; 4 Fundació Puigvert Barcelona Department of Andrology Spain Department of Andrology, Fundació Puigvert Barcelona, Spain; 5 Director Healthy Pleasure Lab London United Kingdom Spain Director Healthy Pleasure Lab London United Kingdom. Spain; 6 Hospital Clinic de Barcelona Department of Urology Barcelona Spain Department of Urology, Hospital Clinic de Barcelona, Barcelona, Spain; 7 Member of Healthy Pleasure Lab Madrid Spain Member of Healthy Pleasure Lab. Madrid, Spain

**Keywords:** Sexual Behavior, COVID-19 [Supplementary Concept], Pandemics

## Abstract

The COVID-19 pandemic has radically changed the way of life around the World. The state of alarm has forced the population to stay at home, radically changing both interpersonal and partner relationships; work at home, social distancing, the continued presence of children at home, fear of infection and not being able to physically meet with others have changed most people's sexual habits. We conducted a review by exploring the impact of the COVID-19 pandemic on sexual behavior in the population from three different countries: Iran, Italy and Spain from each country's perspective. The impact of the coronavirus will be very important in the sexual life of the people and we will attend in the next months or years, to some changes in the relationships at all the levels. The pandemic will negatively affect sexual behaviors due to multiple contact restrictions. In the future, we will be able to assess these effects in more detail.

## INTRODUCTION

Sexual relations suffered a serious blow due to the world pandemic from SARS-Cov-2 (1). COVID-19 has radically changed social relations in the World, both because of the restrictions imposed by the various States and because of the feeling of fear of the contagion that has swept the general population. These changes overwhelmed us in a very short period of time, without leaving time for our mind and bodies to get used to the new situation. The anguish about the world situation, together with the continuous exposure to images of disease and death, has severely tested the emotional stability of each person. In the literature, various studies looked at the general population's psychosocial responses to the severe acute respiratory syndrome epidemic. The topics in the psychological responses included anxiety, fears, depression, anger, guilt, pain and loss, post-traumatic stress and stigma (2). The upheaval of the daily routine, the limitation of everyone's freedom and independence, and the loss of that sense of utility, proper to contributing to community work, have instilled in man a sense of helplessness and loss. From this it is clear how the psychological implications have been devastating and certainly the sexual sphere is the one that has been most affected (3). The fear of contagion itself has therefore reduced physical contact within couples, from simple kissing to full sexual intercourse. The state of constraint to which we were forced to live, side by side for 24 hours a day, the limitation of one's own space and the obligation to share every moment of the day, in some cases exacerbated the quarrels within the cohabiting couple, it exacerbated the differences of opinion, thus weakening the couple bond. Negative emotions are known to negatively affect sexual intercourse. In fact, sexual and reproductive health is a state of physical, emotional, mental, and social wellbeing in relation to all aspects of sexuality and reproduction, not merely the absence of disease, dysfunction, or infirmity. Furthermore now, with many countries in lockdown, sexual habits can also vary significantly (4). Therefore, a positive approach to sexuality and reproduction should recognise the part played by pleasurable sexual relationships, trust, and communication in the promotion of self-esteem and overall wellbeing (5). So COVID-19 has had a negative impact not only in terms of affectivity but also in terms of sexual relationship. In relations between cohabitants, sexual intercourse was affected by the continuous presence of children in the home, given the closure of schools, with the difficulty of finding a moment of intimacy. Sexuality is also influenced by the sense of desire for the other. Psychological factors, specific mood states can inhibit sexual desire. Depression and anxiety have been mostly associated with low levels of desire (6, 7). Those who live a stable relationship but are not living together, on the contrary, have a strong desire for the other, who however cannot be satisfied for the physical distance and the impossibility of approaching due to the restriction in the movements of people imposed by the State. In this case, sexuality can be experienced differently thanks to the use of the Internet, but not all couples are willing to have sex online. As for singles, it is clear how, at a time when social relationships are zeroed, it is difficult to be able to undertake occasional sexual relations, given that there is no opportunity to meet the partner. It should be noted that even the sexual relations between colleagues at work no longer have an opportunity to exist, for those workers whose companies have been closed and smart working has been adopted.

Finally, extra-marital sexual relations are made difficult by the inability to move and reach the home of others due to the legal restrictions imposed by their country of origin, but also by the fact that the respective cohabitants are at home, therefore with the inability to find a suitable place for sexual intercourse. So, if on one hand the psychological implications make the execution of the sexual act less desirable, on the other hand also logistical problems reduce the possibility of having a sexual relationship. When the state of alert is over, a lot of work will have to be done, especially on the couple, to return to normal.

### SARS-CoV2-Transmission and the Sex

In terms of risk and transmission of SARS--CoV-2 during sex, some studies have showed that, the largest amount of virus is present in saliva and, so kissing, a very common practice during sexual intercourse at the pandemic times, is very risky. Furthermore, we should also consider a fecal-oral transmission has been detected in stool samples of infected patients (8, 9).

There is no evidence that the COVID-19 can be transmitted via either vaginal or anal intercourse. There is also evidence of oral-fecal transmission of the COVID-19 and that implies that anilingus may represent a risk for infection. For homosexuals spread from anal intercourses εt oral-fecal way is possible. The pregnant infected women who had vaginal delivery did not have infected babies, so trans vaginal involvement did not seem.

SARS-CoV-2 can be present in the semen of patients with COVID-19, and SARS-CoV-2 may still be detected in the semen of recovering patients. Owing to the imperfect blood-testes/deferens/epididymis barriers, SARS-CoV-2 might be seeded to the male reproductive tract, especially in the presence of systemic local inflammation. Even if the virus cannot replicate in the male reproductive system, it may persist, possibly resulting from the privileged immunity of testes. If it could be proved that SARS-CoV-2 can be transmitted sexually in future studies, sexual transmission might be a critical part of the prevention of transmission, especially considering the fact that SARS-CoV-2 was detected in the semen of recovering patients. Abstinence or condom use might be considered as preventive means for these patients (10).

### Sexual Health during the Pandemic

**Physical Benefits:** There are indications that sexual activity is an integral contributor to quality of life and overall physical health. It has long been understood that poor health can affect sexuality. Diabetes, chronic pain, depression, heart disease and cancer are all examples of conditions that can impair most areas of sexual function.

In pandemic times, management interventions including prolonged periods of quarantine, social distancing, and home confinement, have all-pervasive effects on social and economic life. Regrettably, little information and attention is focused on maintaining sexual health, despite its powerful effect on the overall quality of life in the short and long-term.

**Psychological Benefits:** WHO defines mental health as “a state of complete physical, mental and social well-being” and not merely “the absence of disease or infirmity.” Regarding pandemic periods, mental health is an extremely essential issue that should be noted (11, 12). According to the literature, the most prevalent symptoms in those who have been quarantined are depressed mood, irritability, fear, nervousness, and guilt (13, 14). Scientific evidence has shown a strong link between mental and physical health. Daily activities such as sexual practices are highly related to a person's quality of life and mental health. The negative psychological effects like depressed mood, irritability, fear, nervousness, and guilt during this period are not surprising (2, 16). Other studies have also demonstrated a positive association between duration of quarantine and worse mental health, more specifically symptoms of post-traumatic stress, (PTS) avoidance behaviors and anger (15).

Another key condition is the frustration/boredom of confinement, loss of routine, and social and physical contacts, which seems to be exacerbated when it is not possible to carry out daily activities or to participate in social networking activities.

The long-term effects also appear to be problematic. According to a study carried out with a group of individuals who were quarantined for potential contact with SARS-CoV-2 in the weeks after the quarantine period, a significant percentage of individuals continued to avoid others who were coughing or sneezing, closed places with clusters of people and public spaces. On that note, it is essential to reduce boredom, enhance communication and to activate social contacts, since the impossibility of doing so is a cause not only of immediate anxiety, but also of long-term distress (16).

Sexual health is essential for global health and well-being of individuals, couples and families. Studies correlate sex with increased satisfaction with one's mental health, increased levels of trust, intimacy, and love in relationships, improved ability to perceive, identify, and express emotions and lessened use of immature psychological defense mechanisms (17).

To conclude, the psychosocial and economic implications of the current pandemic and the impact they have on collective, dyadic, and individual adjustment, are expected to have deleterious collateral effects on general health.

**Sexual desire and desire discrepancies:** Concerns around low sexual desire are highly prevalent across populations, ranging from 10-40% and are one of the most widespread sexual problems adults face (18). However, sexual desire discrepancy (when partners report significantly different desires for sexual intimacy) remains one of the most common reasons for couples to seek therapy services due to the negative impact on relationship and sexual satisfaction.

### Erectile Dysfunction

Erectile Dysfunction (ED) is the most common male sexual health concern, affecting between 13-28% of men aged 40-80 years (19), with prevalence increasing with age. While there is no data that explores the relationship between COVID-19 and the additional risk of developing ED, men at greatest risk for having serious complications secondary to COVID-19 are also those traditionally at risk for ED: older adult, diabetic, men with cardiovascular disease, overweight/obese, and with multiple comorbidities (20). Therefore, it is important to consider the role of added stress, anxiety, and physical health implications for men with ED amid the COVID-19 pandemic.

It is not clear, if that COVID-19 may add to the collective risk of developing ED or exacerbate existing ED in men who contract COVID-19; there are previous examples of viral respiratory infections complicated with fibrosis (21). Chronic lung diseases, namely interstitial lung diseases and chronic obstructive pulmonary diseases (COPD) have been associated with ED. In conclusion, despite lack of research on the topic, we may expect ED to worsen during the highly stressful situation men face during the current pandemic. Postponement of most elective, non-urgent medical treatments and putting “on hold” topics that are not a direct, immediate threat to one's health and safety may have a negative impact also on men's sexual health.

These days quarantine time affect on mood of all people and decrease libido and sex because of bored at home, but in another side, since people are staying home without job, they need to have sex with their partner. Since sex relationship affects to immune system, and boost immunity against virus infection, avoiding sex is not recommended.

### Dating and Sexual Activity during the Pandemic

Physical distance is necessary to control the pandemic, so physical dating have disappeared. It is natural that increased levels of stress can reduce this urge, but social distancing and stressful circumstances also increase the need for emotional bonding. We restructured and concluded here important issues to remember during pandemic for maintaining safe and pleasant sexual activity:

### Sex life during the pandemic

Undoubtedly, a new era is present, and we must prepare for the different pandemic and post-pandemic scenarios. The truth is that we still have more questions than answers, and we are currently in the middle of COVID-19 pandemic, a true fact: There isn't any scientific data yet on how this might impact people's sexual and relational lives.

But we can go back to the historical data to help us raise those possible scenarios. Perhaps it can be a window to recreate campaigns during the years of HIV epidemic, making safe sex sexy or could be an open window to create an “immune passport” for coronavirus and let us take advantage to de-stigmatize STD (Sexual Transmitted Diseases) screenings and including them in this type of documents to establish safer sex and not only sex free from coronavirus. So, the main effect is that currently, our society, need to incorporate new COVID-19 sex status.

But, it seems that current debate in the community which deals with sexual health is related to whether there will be an increase in sexual activity and therefore create a new baby boom, or the opposite, the acute anxiety that a crisis supposes and the uncertainty in front of a real and global threat against life, added to the grief for many losses, may would be an erotic killer, and produce more depression and less sex. But even in a society under survival mode, sexuality has a space, because it is a fundamental expression of the human experience. In the broad picture, many people are in lockdown with limited outside activities, staying at home is an opportunity for physical intimacy, assuming you have a live-in sexual partner, of course and be more creative in developing tools for intimacy it's always an option. The main issue here, it is about how to maintain a safe intimacy during and after pandemic times, keeping the adventure and pleasurable feelings at same time alive.

For sure, a new rule has arrived and many forms of intimacy require a closer distance than the six feet of separation recommended by the Centers for Disease Control and Prevention (CDC) (22). But our current digital culture is well-positioned to facilitate shifting models of sexual interaction. Allowing society to have the chance to choose being more resourceful which means a great time to be mindful of our sexual health, which has proven benefits beyond pleasure. But we know that women and men sexual response have different drivers and different response model, one more circular, female and the other more lineal. But the immediate effects in people sex life, independently of their status or gender would be to try to keep safety over pleasure aside, from figure out how to maintain social connection without breaking the guidelines.

And how to reorder pleasure, intimacy and sexual activities with a partner when mandates requires avoiding sex contact and when your safest sex partner, after yourself, is someone you live with according to NYC health department (23). It seems pretty challenging. Even more in our western society, where sexuality has being framed more into an efficiency and accomplishment model than pleasurable, creative and imaginative one. We live in a society where there is a common belief, a cultural and interpersonal expectation that sex should always be “amazing” and with a “perfect” performance. When this is not the case, we consider it inappropriate and understand it as a symptom of “lack of love or affection” or even as a defect in the relationship (24). So, enhance no physical interaction could lead to a new way of sexual behaviour, motivators and triggers for well function or dysfunction, as well. Maybe this new normality could shift models of sexual interaction and we will have the need to research on this new intimacy and sexual behavior for both genders and regardless of their relationship status.

By the moments, people need to be aware, that some may have the virus and not yet have symptoms during the early incubation phase (22) and it could be an issue for who are single and in the active search for a partner, during pandemic avoiding physical contact is what corresponds and then it will be necessary to take measures according to the new findings. Additionally, some people never develop obvious symptoms. For this group, masturbation, sexting phone sex with a partner who doesn't live with them, and intimate devices (used just by the holder person) could enhance self-eroticism and lead men and women to new ways of self-exploration if the interest and sexual desire allows to those activities.

And for those who have sexual partners and have not being exposed to COVID-19 and are healthy, practicing social distancing and have had no known exposure to anyone with COVID-19, sex contact, kissing and touching are more likely to be safe as sharing same space or bed should not be an issue.

The role of fears and anxiety and obsessiveness on sexual dysfunction in quarantine time

The need to feel safe and adventurous at same time during quarantine can be complicated, in fact it is one of the challenges for long-term relationships couples to maintain healthy interest and sexual desire alive in regular “non-quarantine” conditions. We are submerged into an anti-erotic culture resulting from the obsession and optimization for efficiency and perfectionism of sexual performance and this explains much of the low fulfillment of expectations around sexuality (26). In addition, we must take into account, not only the fear and uncertainty existing around how to keep a pleasant and safe sexual activity in quarantine, but also the uncertainty about definitive data regarding fertility, vertical transmission and sexual transmission of COVID-19.

Fear, guilt and anxiety are part of the limiting emotions for a fluid and pleasant sexual response and explain the appearance of some of male and female psychogenic sexual dysfunctions. Especially the well-known performance anxiety.

And what we expect is to find a variability in sexual manifestations in front of chaos and that they would be even dynamic and changing according to how the situation transitions, from the pandemic acute phase to reordering our life by living with COVID-19. So, for those men and women with higher levels of sexual inhibition and lower levels of sexual excitation will find them more vulnerable to sexual response alteration during this time (26, 27).

Maybe it is too soon to forecast an increment for sexual dysfunction or an improvement of sexual function due to fear or anxiety, because human sexuality is thus a complex phenomenon with many contributing factors, from the psychological to social to the biological and there are as many sexualities as there are humans.

### Sexual activity during the COVID crisis- an ongoing survey

Due to the limited information currently available regarding sexual activity during the COVID-19 pandemic, Garcia Cruz and Peraza have create a survey in English and in Spanish version with the objective to explore the sexual behavior during the COVID-19 Pandemic in terms of sexual intercourse, masturbation and sexting; it was conducted from March to April 2020. and distributed via social media (Twitter, LinkedIn and Facebook). A total of 279 (100%) answers were obtained with 58% women, 40% men, 1 transsexual man, 1 transsexual woman and 3 people who labelled themselves as “other”.

#### Sexual intercourse

García-Cruz and Peraza's preliminary results showed out that sexual intercourse has not been affected (less frequent in 31%, same frequent in 41% and more frequent in 14%, no couple 15% in English speaking population report; when was compared the Spanish speaking population: less frequent in 23%, same frequent in 39% and more frequent in 7%, no couple 30%). Surprisingly, a total of 3.2% vs 9.7% in the Spanish and the English population respectively had sexual relationship with different people from their partner during the quarantine.

#### Masturbation

García-Cruz and Peraza survey also addressed this topic, finding that only 10% of the surveyed people considered themselves to be practicing more masturbation during the lockdown. In this specific matter, our survey pointed that 16% of the survey was using chats and social media for sexting and another 5,5% dating apps.

The amount of spare time, the theoretical lack of intimacy with other people and the stress generated by the situation might have led to a rise in masturbation. Besides, as commented above, a significant rise in porn consumption has taken place. The lack of intimacy and the general concern about the global situation can be offered as an explanation for this finding.

#### Sexting

The results of the survey showed that sexual communication via digital strategies would be a good alternative to maintain a certain level of sexual activity.

Although we have been advised to limit social interaction, it is hard to believe that sexually active couple have fully full fit the request of absolute limitation of intimacy. On the other hand, the closure of educational system has led to a lack of intimacy that, together with the general concern and negative thoughts about the present and future situation, might lead to a diminish of sexual drive and activity.

## INCREASE IN PORNOGRAPHY

One of the few sectors that has been benefited from the coronavirus pandemic has been the pornography websites that have experienced meteoric growth. The state of alarm has forced the population to stay at home, radically changing both interpersonal and partner relationships; work at home, social distancing, the continued presence of children at home, fear of infection and not being able to physically meet with others have changed most people's sexual habits.

The consumption of pornography reflects this new situation as reflected in the statistics of Pornhub (28), one of the leading pornography portals worldwide, which has published data on this substantial increase in visits to its website. During this period, this website offered free access to its premium version to everyone to encourage the importance of staying at home and practicing social distancing. Pornhub was founded in 2007 and has more than 120 million visitors per day with an average of 100 billion video views per year. The website receives 36 billion visits per year.

### Worldwide traffic to Pornhub

Worldwide traffic to Pornhub skyrocketed compared to the situation before the pandemic in February 2020 with an elevation that reached its peak increase of 24.4% happened on March 25th after it offered its free premium site to encourage people to stay indoors and distance themselves socially.

### Weekly Traffic Changes

Italy was the first European country to close its borders and put into effect a nation-wide quarantine. The following chart shows how Italy's traffic changed over the last few weeks. The drastic increase of 57% in March 12th, 2020, came after Pornhub offered free Premium service to all of Italy. The same offer of free Pornhub Premium was made beginning in March 16th to the countries of Spain and France. Traffic from Spain increased 61.3% (28).

### Hourly Traffic Changes

Traffic on March 17th was up all over the World, but we can easily see what times the most significant changes had happened when compared to an average day. The largest increase of 31.5% happened in the early morning around 3am. We can surmise that people stayed up later because they didn't need to go to work in the morning and slept in a little longer. Traffic at 1pm was 26.4% higher than normal when people may otherwise be at work (Table-1).

**Table 1:** Worldwide Traffic to Pornhub.

World Hour Traffic Changes	Traffic Increase	Hour
March 17th	31,5%	3 am
	26,4%	1 pm
Italy Hour Traffic Changes		
	47%	2 am
March 11th	25%	5 am
	12%	9pm
Spain Hour Traffic Changes		
March 12th	10,1%	3 am
	6,5%	7 pm

As most of the country was required to stay inside, major changes were observed in Italy's hourly traffic. On March 11th, traffic at 2am was 47% higher than normal and remained 25% above average even at 5am. Evening traffic at 9pm was 12% higher than 9pm on an average day.

Traffic from Spain was 6.1% higher than normal on March 12th. After midnight, traffic increased up to 10.1% at 3am. Early morning traffic was much lower than average, followed by a slight increase in the afternoon and a 6.5% increase at 7pm (27).

## DIVORCES

How the pandemic situation may affect relationships will have to be studied in the future. The pandemic is radically changing couple and sexual relationships: confinement, difficulty in having sex, loss of work, economic problems and an uncertain future can act as triggers to break up many couples. Many weddings have been postponed, and if previous relationship problems already existed, the confinement situation may accelerate this process. In China, where the coronavirus has forced millions of people into isolation, the number of divorce applications has soared in recent months in the provinces most affected, according to local newspapers in these provinces. In Hong Kong's general population in 2004, divorce applications were 21% higher than 2002 levels. The psychological and economic impact of this pandemic will last for months after the return to normal.

## CONCLUSIONS

The truth is that we still have more questions than answers. In the upcoming months and years, we will be able to assess these effects in more detail, but we are sure that COVID-19 will have a negative impact not only in terms of affectivity but also in terms of sexual relationships. The impact of the coronavirus will be very important in the sexual life of the people and we will attend in the next months or years, to some changes in the relationships at all the levels.

Psychological, social and the biological factors should be investigated regarding an increment of sexual dysfunction due to fear or anxiety, since human sexuality is a complex phenomenon with many contributing factors.

General recommendations that can be made are that starting a new relationship is so risky because maybe one of them is infected and having non-monogamous sex also is risky. The only safe way is having sex with primary or monogamous sex, if one of them do not go outside or have a risky job.

On the other hand, the consumption of pornography reflects this new situation with data that show a substantial increase in visits to those websites. The pandemic is radically changing couple and their relationships: confinement, difficulty in having sex, loss of work, economic problems and an uncertain future can act as triggers to break up many marriages. The psychological and economic impact of this pandemic will last for months after the return to normal.
